# A chimeric antigen receptor-based cellular safeguard mechanism for selective *in vivo* depletion of engineered T cells

**DOI:** 10.3389/fimmu.2023.1268698

**Published:** 2024-01-11

**Authors:** Mortimer Svec, Sarah Dötsch, Linda Warmuth, Manuel Trebo, Simon Fräßle, Stanley R. Riddell, Ulrich Jäger, Elvira D’Ippolito, Dirk H. Busch

**Affiliations:** ^1^ Institute for Medical Microbiology, Immunology and Hygiene, School of Medicine and Health, Technical University of Munich, Munich, Germany; ^2^ Translational Sciences and Therapeutics, Fred Hutchinson Cancer Research Center, Seattle, WA, United States; ^3^ Department of Medicine I, Division of Hematology and Hemostaseology, Medical University of Vienna, Vienna, Austria

**Keywords:** chimeric antigen receptor, safeguard mechanism, side effects, on-target/off-tumor, B cell aplasia 2

## Abstract

Adoptive immunotherapy based on chimeric antigen receptor (CAR)-engineered T cells has exhibited impressive clinical efficacy in treating B-cell malignancies. However, the potency of CAR-T cells carriethe potential for significant on-target/off-tumor toxicities when target antigens are shared with healthy cells, necessitating the development of complementary safety measures. In this context, there is a need to selectively eliminate therapeutically administered CAR-T cells, especially to revert long-term CAR-T cell-related side effects. To address this, we have developed an effective cellular-based safety mechanism to specifically target and eliminate the transferred CAR-T cells. As proof-of-principle, we have designed a secondary CAR (*anti*-CAR CAR) capable of recognizing a short peptide sequence (Strep-tag II) incorporated into the hinge domain of an *anti*-CD19 CAR. In *in vitro* experiments, these *anti*-CAR CAR-T cells have demonstrated antigen-specific cytokine release and cytotoxicity when co-cultured with *anti*-CD19 CAR-T cells. Moreover, in both immunocompromised and immunocompetent mice, we observed the successful depletion of *anti*-CD19 CAR-T cells when administered concurrently with *anti*-CAR CAR-T cells. We have also demonstrated the efficacy of this safeguard mechanism in a clinically relevant animal model of B-cell aplasia induced by CD19 CAR treatment, where this side effect was reversed upon *anti*-CAR CAR-T cells infusion. Notably, efficient B-cell recovery occurred even in the absence of any pre-conditioning regimens prior *anti*-CAR CAR-T cells transfer, thus enhancing its practical applicability. In summary, we developed a robust cellular safeguard system for selective *in vivo* elimination of engineered T cells, offering a promising solution to address CAR-T cell-related on-target/off-tumor toxicities.

## 1 Introduction

The use of tumor-specific transgenic receptors to engineer autologous patient-derived T cells and harness them against host cancer cells has opened promising new therapeutic options (1, 2). More importantly, it has allowed the manufacturing of well-characterized T-cell products with highly defined specificity and functionality. Remarkable success has been shown with chimeric antigen receptors (CARs) targeting CD19-expressing refractory and relapsed B-cell malignancies, where the adoptive transfer of CAR-T cells led to durable cancer regression (3–5). Currently, most efforts focus on identifying suitable target/CAR combinations for other malignancies (6) and to increase the specificity, sensitivity and durability of CAR-T cells by a variety of engineering approaches like supporting cytokine production, gene knockouts or smart logic gating (7–9).

However, CAR-T cell therapy also comes with some safety concerns. Short-term side effects like cytokine release syndrome (CRS) (10) and neurotoxicity, termed CAR-T cell-related encephalopathy syndrome (CRES) (11) still occur at high frequencies (5, 12). While these acute toxicities are usually reversible and can be alleviated by immediate immunosuppression by corticosteroids or *anti-*IL-6 receptor antibodies (13), there is still a need for safeguard mechanisms that are truly specific for the transferred cells and mediate the elimination of the cell product within early and - maybe more relevant - late stages after the transfer. Indeed, relevant on-target/off-tumor long-term side effects experienced by patients receiving *anti-*CD19 CAR-T cell therapy are persistent B-cell aplasia and consequent hypogammaglobulinemia (14). Substitution with polyclonal antibodies is an established therapy, but there is no consensus on the proper management of this toxicity (15). Moreover, there is still scarce evidence on the consequences of prolonged B-cell aplasia, such as increased risk of infections and mortality. This becomes more important in children and young adults where the development of long-lived plasma cells resistant to *anti-*CD19 CAR-T cell therapy (16), which could provide a long-lasting humoral response, might still be incomplete.

Besides novel engineering approaches attempting to reduce off-target toxicities by controlling the expression and activity of CARs in terms of timing and dosage (17, 18), there are two main concepts of safety regulation. These are based on the use of suicide genes or cell surface markers that aim at the eradication of the adoptively transferred cells. The predominant suicide genes are the inducible caspase 9 (iCas9) (19) and the herpes simplex virus thymidine kinase (HSV-TK) (20). These suicide genes were sufficient under certain circumstances but showed some drawbacks. In the case of the HSV-TK, the clearance of target cells was incomplete; moreover, immune responses against the transgene - also in immunocompromised patients - limited the *in vivo* persistence of the therapeutic cells after transfer (21). Immunogenicity was less prevalent for the highly humanized iCasp9 (22). Still, this mechanism relied on a high expression level of the iCasp9, and some degree of spontaneous activation triggering auto-apoptosis could not be avoided entirely (19).

Regarding safeguard mechanisms based on surface molecules, one relevant marker is a truncated version of the human epidermal growth factor receptor (EGFRt) that lacks the intracellular domain and is thus physiologically inert (23). We, among others, have shown in syngeneic preclinical models that transferred EGFRt-expressing CAR-T cells could be efficiently depleted upon administration of the monoclonal *anti-*EGFR antibody Cetuximab (24) and were long-term maintained (25), hinting against immunogenicity. Nevertheless, this marker also requires a high surface expression to deliver a complete clearance of transferred cells (24). Despite ongoing improvements towards an optimized EGFRt expression (26), one should still be cautious about the on-target side effects of Cetuximab. The other caveat is the dependence of this safety mechanism on the antibody-dependent cellular cytotoxicity of the patient.

In this study, we propose a cellular safeguard mechanism that exploits the highly specific antigen recognition and potency of a CAR-T cell capable of recognizing a tag within a different target CAR-T cell. We could show that these so-called ‘*anti-*CAR CAR-T cells’ exhibit potent cytotoxicity *in vitro* as well as *in vivo*, including a clinically relevant mouse model that mirrors established long-term CAR-T cell-related on-target/off-tumor toxicity. In this context, adoptively transferred *anti-*CAR CAR-T cells reversed persistent B-cell aplasia induced by *anti-*CD19 CAR-T cells. Intriguingly, high doses of *anti-*CAR CAR-T cells could mediate this effect also in the absence of pre-conditioning prior to cell transfer.

## 2 Materials and methods

### 2.1 Animal models and cell lines

C57BL/6 mice (female, 8-11 weeks old) were acquired from Envigo. RAG^-/-^ mice and C57BL/6 mice on different congenic backgrounds (CD45.2^+/+^, CD45.1^+/+^, CD45.1^+/-^, CD90.1^+/+^, CD90.1^+/-^) were derived from in-house breeding under specific pathogen-free conditions at our mouse facility at the Technical University Munich, Institute for Medical Microbiology, Immunology and Hygiene. The performed animal experiments were approved by the district government of Upper Bavaria (Department 5—Environment, Health and Consumer Protection ROB- 55.2-2532.Vet_02-17-138).

Murine splenocytes were cultured in RMPI (Life Technologies, Cat #31870074) with 10% fetal bovine serum (FCS) (Sigma, Cat #F7524), 0.025% L-glutamine (Sigma, Cat #G8540), 0.1% HEPES (Roth, Cat #HN77.4), 0.001% gentamycin (LifeTechnologies, Cat #15750037), 0.002% penicillin/streptomycin (LifeTechnologies, Cat #10378016) and 15 ng/ml recombinant human (rh) IL-15 (Peprotech, Cat # 200-15). The Platinum-E packaging cell line (PlatE) was cultured in DMEM (Life Technologies, Cat #10938025) supplemented with 10% FCS, 0.025% L-glutamine, 0.1% HEPES, 0.001% gentamycin, 0.002% streptomycin. All cells were grown in a humidified incubator at 37°C and 5% CO2.

### 2.2 CAR DNA template design

scFvs were generated by fusing the variable regions of the heavy (VH) and light (VL) chains with a short (G4S)_3_ linker. For CAR constructs, the signal peptide of CD8α was followed by the scFv, a hinge domain (a triple repetitive sequence of Strep-tag II (STII) for *anti-*CD19 CAR and a CD8a spacer domain for the *anti-*CAR CAR) and parts of the IgG4-Fc molecule. This extracellular domain was followed by a transmembrane region originated from the CD28 chain and the intracellular signaling domains CD3-zeta. A truncated version of the EGFR (EGFRt) was added at the 5’ and separated from the CAR sequence by a self-splicing viral P2A element. As control, a construct with the same elements as the *anti-*CD19 CAR but without the scFV (defined as ‘mock’) was generated. DNA templates were designed *in silico* and synthesized by GeneArt (Thermo Fisher Scientific) or Twist Bioscience into pMP72 vectors. scFv sequence of *anti-*CAR CAR (clone: 5G2) were kindly provided by the lab of Prof. Riddell.

### 2.3 Retroviral transduction

For the production of retroviral particles, PlatE cells were transiently transfected with a pMP72 expression vector by calcium phosphate precipitation. For this, 15 µl of a 3.31 M MgCl_2_ was mixed with 18 µg vector DNA and filled up to 150 µl with ddH_2_O. This solution was slowly added under vortexing to 150 µl transfection buffer (274 mM NaCl, 9.9 mM KCl, 3.5 mM Na_2_HPO_4_ and 41.9 mM HEPES), and the final transfection mix was added to PlatE cells for 6 h at 37°C, followed by a complete medium exchange. The virus-containing supernatant was harvested 48 and 72 h after transfection by filtration (0,45 µm sterile filter) and stored at 4°C for up to one week. Mouse splenocytes were obtained from CD45/CD90 congenic mice by mashing the spleen into single-cell suspensions. After red cell lysis, splenocytes were stimulated at a concentration of 10^7^ cells/mL with purified anti-mouse CD3 (BD Biosciences, clone 145-2C11, 1:1000, Cat #553058) and anti-mouse CD28 (BD Biosciences, clone 37.51, 1:3000, BD Cat #553295) antibodies in presence of 25 U/ml recombinant human interleukin-2 (Peprotech, Cat #200-02). For *anti*-CAR CAR transduction, splenocytes were previously depleted of B cells by magnetic purification (Miltenyi Biotec, Cat #130-048-701) using an anti-mouse CD19-FITC antibody (BD Biosciences, clone 1D3, 1:100, Cat #553785) according to the manufacturer’s protocol. After 24 h, stimulated splenocytes were collected and transduced via spinoculation. For this, tissue-culture untreated 24-well plates were coated overnight with retronectin (TaKaRa, Cat #T100B), purified anti-mouse CD3 (1:1000) and anti-mouse CD28 (1:3000) antibodies. The virus-containing supernatant was centrifuged onto the plates at 3000 g at 32°C for 2 h. After removal of the supernatant, cells were transferred and centrifuged at 800 g at 32°C for 1.5 h. Cells were then rested for two days before administration to the mice.

### 2.4 Flow cytometry

For *in vitro* assays, cells were harvested from the culture and washed twice with FACS buffer (1x PBS, 0.5% (w/v) bovine serum albumin (BSA), pH 7.45) before staining. For blood analysis, blood was sampled from the tail vein and stored in heparin at room temperature. Bone marrow was acquired from the hind legs by removal of muscles and rinsing with complete RPMI medium whereas lymph nodes and spleens were homogenized into single-cell suspension by mashing. Red cell lysis was performed for single-cell suspensions from all organs and blood with RBC lysis buffer (90% 0.17M NH4Cl in Tris-HCl). After lysis, cells were first stained with anti-CD16/32 antibody (Biolegend, clone 93, 1:400, Cat #101301) for 20 min at 4°C (only cells from lymphoid tissues), followed by surface marker staining for 20 min at 4°C in the dark. In this study we used the following antibodies for surface staining: CD3-APC (Biolegend, clone 145-2C11, 1:100, Cat #100312), CD3-FITC (Life Technologies, clone 17A2, 1:100, Cat #11003282), CD8-PacO (Life Technologies, clone 5H10, 1:50, Cat #MCD0830), CD19-ECD (BD Bioscience, clone 1D3, 1:300, Cat #562291), CD45.1-PacBlue (Life Technologies, clone A20, 1:100, Cat #48045382), CD45.1-APC (Life Technologies, clone A20, 1:100, Cat #17045382), CD90.1-PacBlue (Life Technologies, clone HIS51, 1:500, Cat #48-0900-82), CD90.2-APC (Life Technologies, clone 53-2.1, 1:500, Cat #17090283), EGFR-PE (Biolegend, clone AY13, 1:2000, Cat #352904), PD-1-PE-Cy7 (Life Technologies, clone J43, 1:100, Cat #25-9985-80), TIM3-BV421 (Biolegend, clone RMT3-23, 1:100, Cat #119723), CD69-BV510 (Biolegend, clone H1-2F3, 1:100, Cat #104531). Live/dead discrimination was performed either by addition of propidium iodide (LifeTechnologies, 1:100, Cat #P1304MP) 3 min before the end of the staining or with ethidium-monoazide-bromide (LifeTechnologies, 1:1000, Cat #E1374) for 15 min at 4°C under bright light. Absolute quantification was performed by using 123count eBeads (Life Technologies, Cat #01-1234-42) according to the manufacturer’s instructions. Specific information is further provided for each experiment. Samples were acquired on a CytoFLEX S flow cytometer (Beckman Coulter). All flow cytometry data were analyzed with FlowJo v10.

### 2.5 Antigen-specific *in vitro* stimulation and intracellular cytokine staining

5x10^4^ effector *anti-*CAR CAR-T cells (EGFRt^+^) were co-cultured with target *anti-*CD19 CAR-T cells (EGFRt^+^) at different effector-to-target ratios in 96-well plates. After 1 h co-incubation, 1x GolgiPlug was added (BD PharMingen, Cat #555029) and cells were incubated for additional 4 h at 37°C. 25 ng/ml Phorbol-12-myristat-13-acetate (Sigma, Cat #P1585) and 1 μg/ml ionomycin (Sigma, Cat #I9657) were used as positive controls, while untransduced cells were used as negative controls. After co-culture, staining for live/dead discrimination was performed with ethidium-monoazide-bromide as described before, followed by surface marker antibody staining for congenic markers (CD45.1, CD45.2) and EGFRt for 20 min at 4°C. Cells were permeabilized using Cytofix/Cytoperm (BD Biosciences, reference #554714) and stained intracellularly for TNF-α-PE-Cy7 (BD Biosciences, clone MP6-XT22, 1:100, Cat #557644), INF-γ-APC (LifeTech, clone XGM1.2, 1:400, Cat #17-7311-82) and IL-2 (LifeTech, clone JES6-5H4, 1:100, Cat #12-7021-82). As bulk CAR-T cell products were used, the number of transferred cells always refers to the amount of congenic marker^+^EGFRt^+^ cells. The total amount of transferred cells was defined by flow cytometry according to the expression of the EGFRt marker.

### 2.6 Flow cytometry-based cytotoxic T lymphocyte assay

4x10^4^ effector *anti-*CAR CAR-T cells (EGFRt^+^) were co-cultured with target *anti-*CD19 CAR-T cells (EGFRt^+^) at different effector to target ratios in 96-well U-bottom plate at a concentration of 7.5x10^5^ cells/mL for 48 h. At the beginning of the incubation (0 h), and after 24 and 48 h, 200 µL of the cell suspension was harvested for surface marker antibody staining of congenic markers (CD90.1, CD45.1), CD3, CD8, CD19 and EGFRt. Staining for live/dead discrimination was performed with propidium iodide. As bulk CAR-T cell products were used, the number of transferred cells always refers to the amount of congenic marker^+^ EGFRt^+^ cells. The total amount of transferred cells was defined by flow cytometry according to the expression of the EGFRt marker.

### 2.7 Western blot

Transduced mouse splenocytes were rested for 5 days or activated with anti-CD3 (1:1000) and anti-CD28 (1:3000) antibodies for 24 h prior analyses. Cell lysates were generated by addition of 200 µl 1X SDS sample buffer (62.5 mM Tris pH 6.8, 2% w/v SDS, 10% Glycerol, 0.01% Bromophenol blue, 50 mM DTT) to 2x10^6^ cells, followed by sonication for 10 min and incubation at 95°C for 5 min. The solution was centrifuged for 5 min at 13.000 g, and 15 µl of the supernatant were run on an 8% SDS-PAGE gel (stacking gel: TRIS 0.5 M pH 6.5, 0.4% SDS, 40% Acrylamide, 10% APS TEMED; separating gel: Tris 1.5 M pH 8.8, 0.4% SDS, 40% Acrylamide, 10% APS TEMED) for 1 h at 150 V. The proteins were semi-dry blotted onto a nitrocellulose membrane at 10 V for 30 min. The efficiency of the transfer was evaluated via Ponceau staining (0.5% Ponceau, 1% acetic acid). The membrane was blocked in Tris-buffered saline containing 0.1% Tween (TBS-T buffer) with 5% nonfat dry milk (blocking buffer) for 1 h at room temperature. Afterwards the membrane was stained with anti-pCD3ζ antibody (Life Technologies, clone EM54, 1:3000, Cat #PIMA528538) in TBS-T buffer containing 5% BSA at 4°C. As loading control, GAPDH was stained with anti-GAPDH antibody (Thermo Fisher Scientific, clone 1D4, 1:3000, Cat #MA1-16757). After 12 h, the membrane was washed in TBS-T buffer and incubated with a horseradish peroxidase-conjugated anti-mouse secondary antibody (Cell Signaling, 1:1000, Cat #7076S) in blocking buffer for 1 h at room temperature. After washing, the membrane was incubated with 200 µl enhanced chemiluminescence solution (Biorad, Cat #1705061) for 5 min at room temperature and analyzed with the INTAS chemoluminescence detection system.

### 2.8 Adoptive T cell transfer

For the co-injection model, Rag^-/-^ or C57BL/6 mice were sublethally irradiated (5 Gy) the day before the simultaneous adoptive transfer of 1x10^6^ *anti-*CD19 CAR-T cells and 2x10^6^ *anti-*CAR CAR-T cells by intravenous injection (i.v.). Transferred cells and B cells were followed in the blood at different time points and in bone marrow, spleen and lymph nodes at the endpoint.

For the therapeutic model, 2x10^6^ *anti-*CD19 CAR-T cells were transferred i.v. into sublethally irradiated mice (5 Gy). After 27 days, mice were either or not irradiated with a 2 Gy dose and treated the day after i.v. with 4x10^6^ or 8x10^6^ *anti-*CAR CAR-T cells, or 4x10^6^ mock T cells (CAR construct without the scFv). Moreover, one group received the monoclonal antibody Cetuximab (1mg per mouse) (Bristol-Myers Squibb) by intraperitoneal injection. Transferred cells and B cells were followed in the blood at different time points and in bone marrow, spleen and lymph nodes at the endpoint.

As bulk CAR-T cell products were used, the number of transferred cells always refers to the number of congenic marker^+^EGFRt^+^ cells. The total amount of transferred cells was defined by flow cytometry according to the expression of the EGFRt marker. The specific congenic marker combinations are indicated for each experiment.

### 2.9 Visualization and statistical testing

Data were visualized using GraphPad Prism (V9.1). All statistical tests were performed using GraphPad Prism (V9.1). Specific details regarding statistical analyses can be found in the corresponding figure legends. Levels of significance were defined as the following: ns = p > 0.05, *p ≤ 0.05, **p ≤ 0.01, ***p ≤ 0.001, ****p ≤ 0.0001. Data are presented as mean ± standard deviation or mean + standard deviation, as indicated in figure legends.

## 3 Results

### 3.1 Design and *in vitro* functionality of *anti-*CAR CAR-T cells

**Figure 1:**
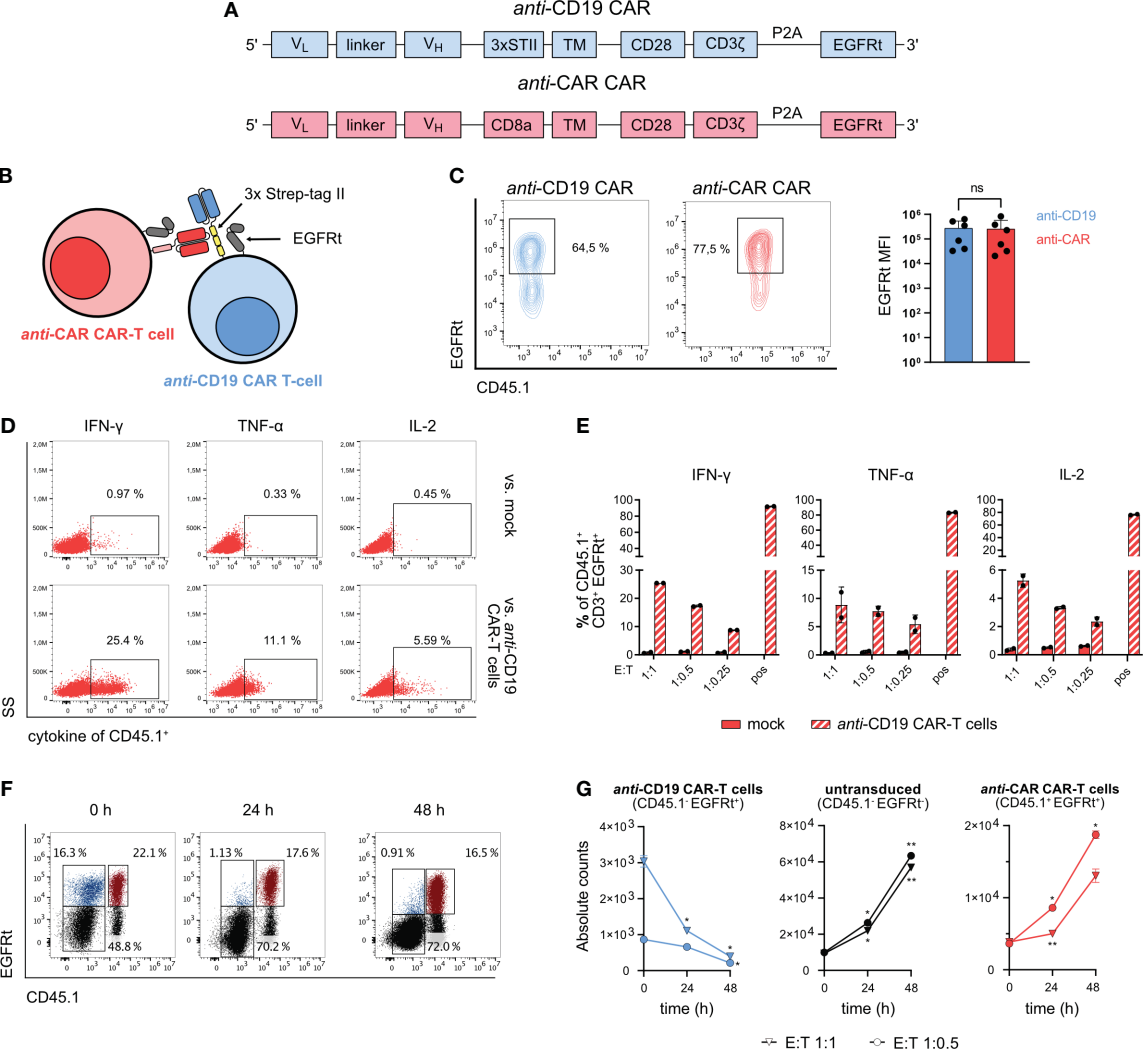
Design and *in vitro* functionality of *anti-*CAR CAR-T cells. **(A)** Schematic of the CAR sequences for retroviral transduction. **(B)** Schematic of the mechanism of action of *anti-*CAR CAR-T cells. **(C)** Cell surface expression of CAR constructs after retroviral transduction measured by EGFRt co-expression. **(D, E)** 5x10^4^ *anti-*CAR CAR-T cells were co-cultured with B cell-depleted *anti-*CD19 CAR-T cells or untransduced cells at the indicated E:T ratios for 4 h. Representative flow cytometry plots (E:T 1:1) **(D)** and quantification **(F)** of intracellular staining of IFN-γ, TNF-α and IL-2. Pregated on CD3^+^ EGFRt^+^ living lymphocytes. **(F, G)** 4x10^4^ *anti-*CAR CAR-T cells (red: CD45.1^+^ EGFRt^+^) were co-cultured with B cell-depleted bulk *anti-*CD19 CAR-T cells (blue: CD45.1^-^ EGFRt^+^; gray: CD45.1^-^ EGFRt^-^) at the indicated E:T ratios and analyzed at 0, 24 and 48 h. Representative flow cytometry plots **(G)** and quantification **(H)** of the co-culture killing assay. Pregated on CD3^+^ living lymphocytes. Data are shown as mean + SD in **(C)** and mean ± SD in **(E, G)**. In **(C)**, statistical analyses have been performed by Mann-Whitney nonparametric test. In **(G)** statistical analyses have been performed by two-way ANOVA with Dunnett’s multiple comparisons test using 0 h as reference. ns = p > 0.05, *p < 0.05, **p < 0.01.

In this study, we used a fully syngeneic second-generation *anti-*CD19 CAR as the primary ‘therapeutic’ CAR. We further included in the hinge region a sequence containing a three-time in tandem repetition of an 8 amino acid peptide, named Strep-tag II (27). Previous investigations have demonstrated that the inclusion of this triple Strep-tag II in the CAR construct does not compromise the functionality of the corresponding engineered T cells (28). To target *anti-*CD19 CAR-T cells, we generated a second CAR construct where the scFv could specifically recognize the Strep-tag II. This CAR, referred to as ‘*anti-*CAR’ CAR from now on, was additionally equipped with the same CD3zeta/CD28 intracellular domains and truncated human EGFR (23, 24) as the *anti-*CD19 CAR (**Figure 1A**). This engineering approach theoretically enables the depletion of fully functional *anti-*CD19 CAR-T cells by the second *anti-*CAR CAR-T cells (**Figure 1B**). For appropriate discrimination, the two CARs were always re-expressed in splenocytes from mice harboring distinct CD45/CD90 congenic marker combinations. Furthermore, B cells were depleted from spleen-derived cell suspensions before viral transduction to avoid stimulation of *anti-*CD19 CAR-T cells prior any *in vitro* or *in vivo* assay. Both receptors could be successfully expressed in primary murine splenocytes through retroviral gene delivery and showed comparable surface expression levels (**Figure 1C**).

To demonstrate the *in vitro* functionality of the newly designed *anti-*CAR CAR-T cells, we first examined intracellular cytokine release upon target-specific stimulation during co-culture with Strep-tag II-expressing *anti-*CD19 CAR-T cells. The *anti-*CAR CAR-T cells showed reliable cytokine production (IFN-γ, TNF-α and IL-2) in a dose-dependent manner in presence of the target cells. Moreover, there was little to no unspecific signaling when co-cultured with untransduced control cells (**Figures 1D, E**). For evaluating cytotoxic capacity, we again co-cultured *anti-*CD19 CAR-T cells with *anti-*CAR CAR-T cells at different effector-to-target (E:T) ratios and monitored the absolute number of each T cell population over 48 hours. By using bulk populations, we could also assess the specificity of killing by tracking the survival of unedited (EGFRt^-^) cells. Importantly, while the untransduced control cells without antigen expression, as well as the effector cells, proliferated over time, we observed a significant decrease of the target *anti-*CD19 CAR-T cells (**Figures 1F, G**). Altogether, these data demonstrate the specific target recognition of our *anti-*CAR CAR-T cells, which ultimately translates into effective cytotoxic T-cell functions.

Finally, we investigated whether *anti*-CAR CAR-T cells showed any tonic signaling activity. For this purpose, we firstly analyzed the baseline phosphorylation of CD3zeta in rested splenocytes, using freshly restimulated cells as control. While we observed elevated levels of pCD3z in restimulated *anti*-CAR CAR-T cells, we did not detect any level exceeding those found in control cells in rested splenocytes (**Supplementary Figure 1A**). To confirm this phenotype, we also assessed the persistence and activation state of adoptively transferred *anti*-CAR CAR-T cells under homeostatic conditions (**Supplementary Figure 1B**). We observed overall similar numbers of circulating *anti*-CAR CAR-T cells compared to untransduced mock control cells (**Supplementary Figure 1C**), and, as expected, no influence on the B-cell compartment except for the drop in the first week due to the pre-conditioning regimen prior T-cell transfer (**Supplementary Figure 1D**). Furthermore, we did not detect neither consistent upregulation of activation markers (CD69 and PD-1) nor exhausted cells (PD-1^+^TIM3^+^) (**Supplementary Figures 1E, F**). These observations were confirmed also in *anti*-CAR CAR-T cells infiltrating secondary lymphoid organs, except from the detection of a small fraction of CD69^+^ cells in the bone marrow (**Supplementary Figure 1G**). Altogether, we could not find robust indications of tonic signaling.

### 3.2 *Anti*-CAR CAR-T cells display *in vivo* killing in immunocompromised mice in a model of acute antigen encounter

Our *in vitro* data indicated a certain degree of functionality for the designed *anti-*CAR receptor. However, such evidence could not definitively predict the *in vivo* functionality, as this relies on additional factors such as the overall antigen load, target cell accessibility and the receptor moiety itself, among others (29, 30).

**Figure 2:**
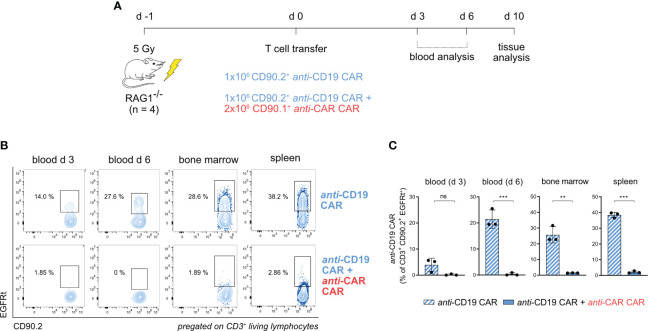
*anti-*CAR CAR-T cells display *in vivo* killing in immunocompromised mice in a model of acute antigen encounter. **(A)** Schematic of the experimental setup. **(B, C)** Representative flow cytometry plots **(B)** and quantification **(C)** of *anti-*CD19 CAR-T cells (EGFRt^+^CD90.2^+^) in the peripheral blood at the indicated time points and in tissues in the presence or not of *anti-*CAR CAR-T cells. Data are shown as mean ± SD. In **(C)**, statistical analyses were performed by unpaired t-test. ns = p > 0.05, **p < 0.01, ***p < 0.001.

To assess the *in vivo* functionality of our *anti-*CAR construct with minimal confounders, we first conducted a short-term *in vivo* killing assay in the imunocompromised RAG1^-/-^ mouse model, which lacks mature T cells. As before, we generated *anti-*CD19 and *anti-*CAR CAR-T cells from different congenically labelled splenocytes, which were simultaneously injected in pre-conditioned mice. We monitored the adoptively transferred cells in the periphery through blood analysis for 6 days and in tissues on day 10 (**Figure 2A**). While *anti-*CD19 CAR-T cells were reliably detected in the blood when transferred alone, we could not detect circulating *anti-*CD19 CAR-T cells when both T-cell products were co-transferred. This observation was consistent in secondary lymphoid tissues (**Figures 2B, C**; **Supplementary Figure 2**), suggesting infiltration properties that are expected from a cell-based mechanism.

### 3.3 Anti-CAR CAR-T cells can restore B cells in CD19 CAR-treated immunocompetent mice

**Figure 3:**
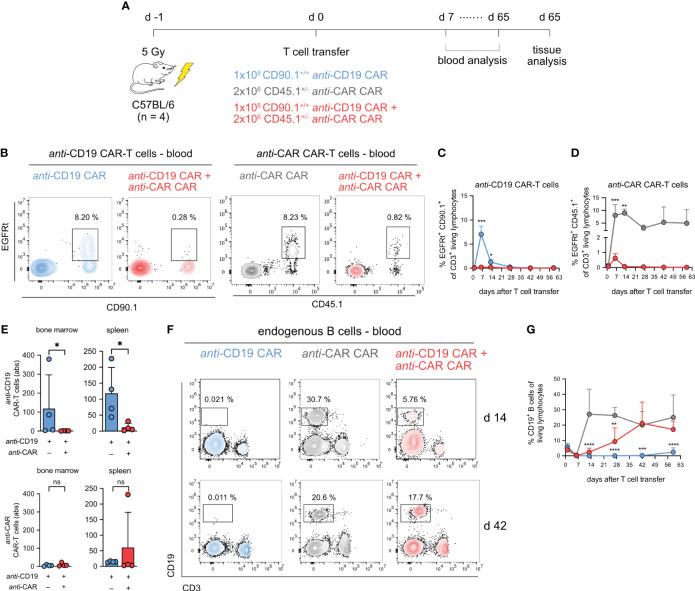
*anti-*CAR CAR-T cells can restore B cells in CD19 CAR-treated immunocompetent mice. **(A)** Schematic of the experimental setup. **(B)** Representative flow cytometry plots of *anti-*CD19 CAR-T cells (EGFRt^+^CD90.1^+^) and *anti-*CAR CAR-T cells (EGFRt^+^CD45.1^+^) at day 6 post infusion according to the different treatment schemes. Cells are pregated on living CD3^+^ T cells. **(C, D)** Quantification of the frequencies of *anti-*CD19 CAR-T cells **(C)** and *anti-*CAR CAR-T cells **(D)** in blood over time. **(E)** Quantification of tissue-infiltrating *anti-*CD19 and *anti*-CAR CAR-T cells. **(F)** Representative flow cytometry plots of living CD19^+^ B cells according to the different treatment schemes at the indicated time points. **(G)** Kinetics of B cells in blood over time. Data are shown as mean + SD. In **(C)** and **(G)** statistical analyses were performed by two-way ANOVA with Sidak’s multiple comparisons test **(C)** and Dunnett’s multiple comparisons test using the *anti*-CAR group as reference **(G)**. In **(E)** statistical analyses were performed with non-parametric Mann-Whitney t test. ns = p > 0.05, *p < 0.05, **p < 0.01, ***p < 0.001, ****p > 0.0001.

As the next step, we setup up a similar experiment using an immunocompetent wild type C57BL/6 mouse model as a recipient. We also included an additional control group treated solely with *anti-*CAR CAR-T cells (**Figure 3A**; **Supplementary Figure 3A**). In this model, the administration of *anti-*CD19 CAR-T cells induces long-lasting B-cell aplasia (31), similar to what reported in patients receiving *anti-*CD19 CAR therapy (5, 32). Thus, this syngeneic model allowed us to monitor the efficacy of *anti-*CAR CAR-T cells in terms of depleting the *anti-*CD19 CAR-T cells as well as reconstituting the B-cell compartment eliminated by the primary therapeutic CAR-T cells (24). Moreover, this model presented challenges more relevant to potential applications in humans, such as the competition with other immune cell populations and, most importantly, a stimulus for the primary *anti-*CD19 CAR, which was still expected to functionally recognize B cells.

Looking at the dynamics of *anti-*CD19 CAR-T cells in the blood (**Supplementary Figure 3B**), we observed a well-known pattern of expansion and contraction within the first two weeks in mice treated solely with the primary CAR-T cells (**Figures 3B, C**; **Supplementary Figure 3C**). This phenomenon is commonly observed in CD19^+^ preclinical tumor models as well as in patients receiving *anti-*CD19 CAR-T cells, and, in our model, was triggered by the endogenous B cells. In sharp contrast, as anticipated from our previous data, this kinetic did not occur in presence of the *anti-*CAR CAR-T cells. Moreover, we did not detect *anti-*CD19 CAR-T cells throughout the observation period, further indicating the persistent functionality of the treatment over time (**Figures 3B, C**; **Supplementary Figure 3C**). While we observed peripheral expansion and contraction of *anti-*CAR CAR-T cells in mice receiving both CAR-T cell types, this occurred to a much lesser extent. The high circulating levels of the *anti-*CAR CAR-T cells when transferred alone, instead, were a sign of T cells with no specificity in the used experimental model (**Figures 3B–D**; **Supplementary Figure 3C**). In secondary lymphoid tissues, we found sustained depletion of *anti-*CD19 CAR-T cells, despite *anti*-CAR CAR-T cells were barely detectable (**Figure 3E**).

We finally investigated the endogenous B-cell compartment, which represents the off-tumor target of our primary CAR-T cells. In the *anti-*CAR-treated control group, B cells recovered within seven days post irradiation, while mice receiving *anti-*CD19 CAR-T cells experienced long-term aplasia, confirming that the addition of the Strep-tag in the hinge region did not affect functionality. Importantly, in mice receiving the combination of the two CAR-engineered T cells, B cells reconstituted early at lower frequency and eventually reached levels comparable to our control group approximately six weeks after infusion (**Figures 3F, G**).

In summary, our findings show that *anti-*CAR CAR-T cells can effectively deplete primary CAR-T cells in an immunocompetent host, leading to the reversal of the unwanted long-term B-cell aplasia induced by *anti-*CD19 CAR-T cells as a side effect.

### 3.4 Therapeutic *anti-*CAR CAR-T cell application reconstitutes B cells also in absence of pre-conditioning treatment

**Figure 4:**
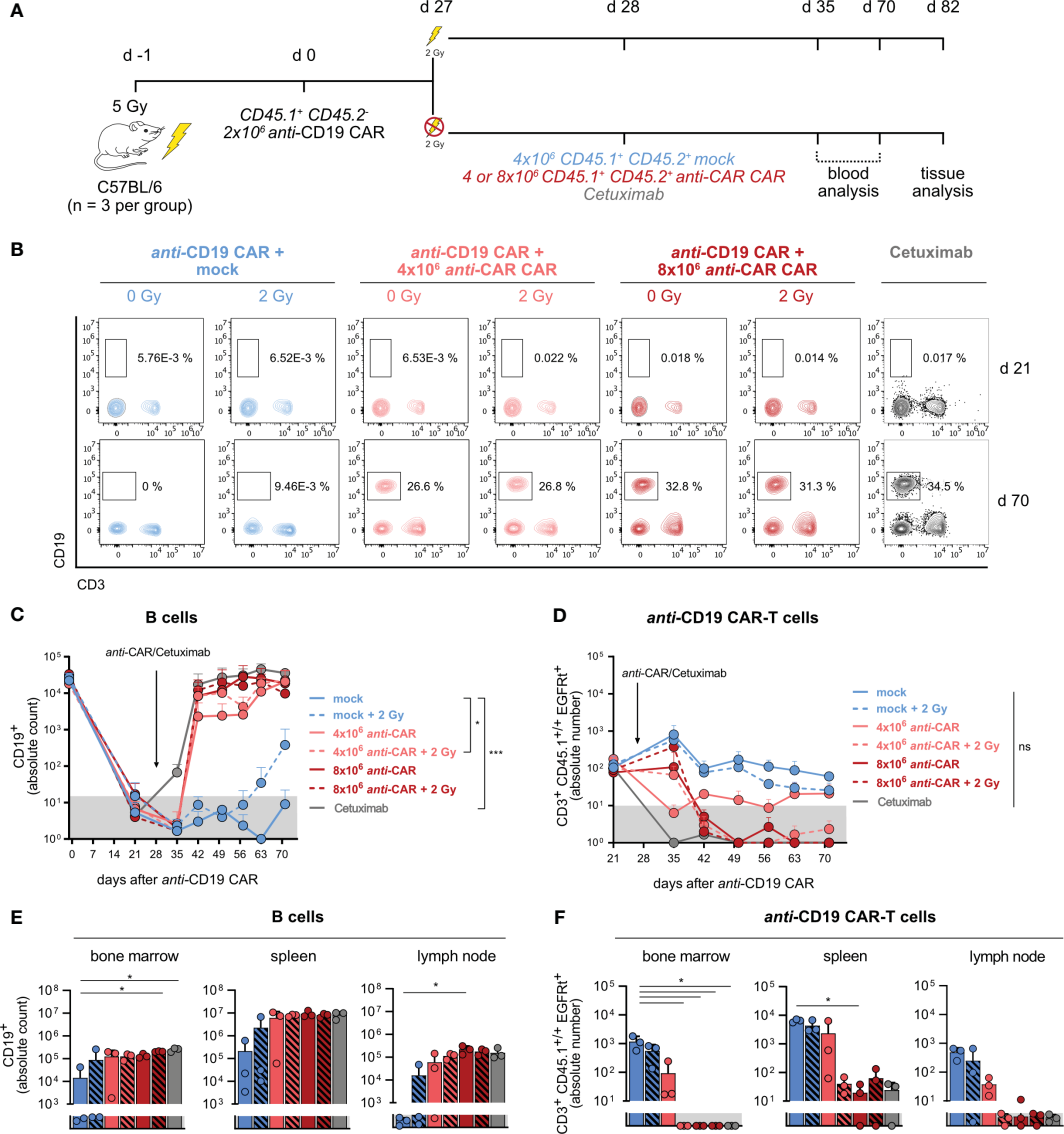
Reliable B-cell reconstitution after therapeutic *anti-*CAR application also in the absence of pre-conditioning treatment. **(A)** Schematic of the experimental setup (n=3). **(B)** Representative flow cytometry plots of B cells one week prior (upper) and six weeks after (lower) *anti-*CAR CAR-T cell transfer. **(C, D)** Quantification of B cells **(C)** and *anti-*CD19 CAR-T cells **(D)** in blood. **(E, F)** Absolute numbers of B cells **(E)** and *anti-*CD19 CAR-T cells **(F)** in secondary lymphoid organs at the endpoint. Data are shown as mean + SD. In **(C, D)**, statistical analyses have been performed by two-way Anova with Dunnett’s multiple comparisons test using the mock group as reference. In **(E, F)**, statistical analyses have been performed by Kruskal-Wallis test with Dunnett’s multiple comparisons test using the mock group as reference. ns = p > 0.05, *p < 0.05, ***p < 0.001.

Our previous data proved the functionality and reliability of the *anti-*CAR approach in a model of simultaneous target engagement. Next, we sought to investigate whether it could effectively reverse a long-term side effect caused by a primary therapeutic CAR-T cell product. To address this question, we induced stable B-cell aplasia in wild-type immunocompetent C57BL/6 mice by administering *anti-*CD19 CAR-T cells, and then transferred *anti-*CAR CAR-T cells four weeks later (**Figure 4A**). Achieving successful engraftment of transferred T cells usually requires lymphodepletion, a step commonly used in clinical practice to enhance the effectiveness of CAR-based therapy in patients (33). Nevertheless, consenting patients for a second round of pre-conditioning, in particular in cases of disease remission, can be challenging. Therefore, we compared the efficiency of *anti-*CAR CAR-T cell transfer with and without pre-conditioning, using different cell doses. Additionally, we included a CAR construct without the scFv (mock) as a negative control, and the monoclonal *anti-*EGFR Cetuximab as a positive control, known for depleting adoptively transferred EGFRt-expressing CAR-T cells and promoting B-cell reconstitution (24) (**Figure 4A**; **Supplementary Figure 4A**).

All mice showed B-cell aplasia until 21 days from *anti-*CD19 CAR-T cell transfer, which was the last time point of monitoring prior to the second T-cell transfer. Mice receiving *anti-*CAR CAR-T cells exhibited reliable B-cell reconstitution as early as two weeks post-administration, reaching levels comparable to Cetuximab-treated mice (**Figures 4B, C**). Noteworthy, there were no significant differences among the four *anti-*CAR-treated groups, except in mice receiving the lower T-cell dose without pre-conditioning. However, despite a slower reconstitution kinetic, B-cell frequencies in this group eventually reached comparable levels with the other groups at later time points. Importantly, mice receiving the control mock CAR-T cells maintained stable B-cell aplasia throughout the observation period also in combination with the second pre-conditioning, except for one mouse (**Figures 4B, C**). This observation suggested that *anti-*CD19 CAR-T cells reliably persisted and remained functional after irradiation. Moreover, it also attributed the B-cell reconstitution to the specific depletion of *anti-*CD19 CAR-T cells by the secondary safeguard CAR-T cells. This was further supported by the clear presence of *anti-*CD19 CAR-T cells in mice receiving the mock CAR-T cells at both early (day 21) and late (day 70) time points, regardless of irradiation, while *anti-*CAR-treated mice showed no detectable *anti-*CD19 CAR-T cells (**Figure 4D**; **Supplementary Figure 4B**).

Similarly to what observed in the peripheral circulation, tissue analysis revealed high B-cell levels and undetectable *anti-*CD19 CAR-T cells also in the spleen, lymph nodes and bone marrow of *anti*-CAR CAR-T cell treated mice. Intriguingly, the group receiving low-dose *anti-*CAR without pre-conditioning showed more variable B-cell levels and persisting infiltrating *anti-*CD19 CAR-T cells (**Figures 4E, F**), despite no detectable B cells in the blood.

**Figure 5:**
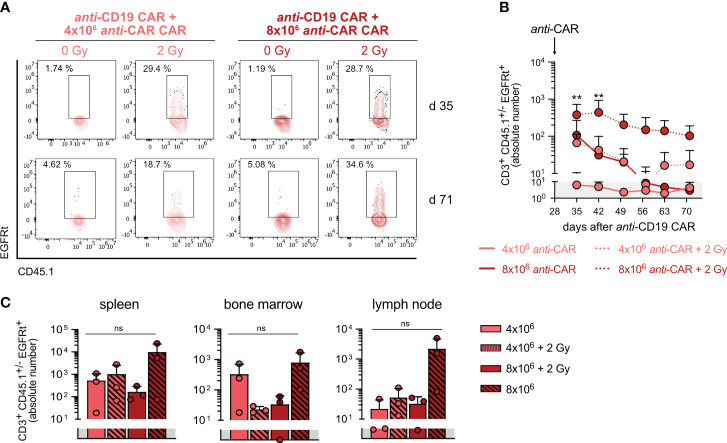
*anti*-CAR CAR-T cells persist also in absence of pre-conditioning. **(A)** Representative flow cytometry plots of *anti-*CAR CAR-T cells one week (upper) and six weeks after (lower) *anti-*CAR treatment. **(B, C)** Quantification of *anti-*CAR CAR-T cells in blood **(B)** and lymphoid tissues at the endpoint **(C)**. Data are shown as mean+SD. Grey area indicates the detection limit. In **(B)** statistical analyses have been performed by two-way Anova with Dunnett’s multiple comparisons test using the 4x10^6^ cells group as reference. In **(C)** statistical analyses have been performed by Kruskal-Wallis test with Dunnett’s multiple comparisons test using the ‘4x10^6^ cells’ group as reference. ns = p > 0.05, **p < 0.01.

We finally examined the persistence of the *anti-*CAR CAR-T cells and observed a clear relationship between the cell dose and pre-conditioning. As expected, irradiation facilitated engraftment of the transferred cells regardless of cell doses. Indeed, circulating *anti-*CAR CAR-T cells remained detectable only in irradiated mice (**Figures 5A, B**), with the highest frequencies observed in mice receiving 8x10^6^ cells plus irradiation in both blood and tissues (**Figure 5**). Intriguingly, the higher cell dose compensated for the absence of pre-conditioning, at least during the initial weeks in the circulation, as indicated by comparable levels between the groups 8x10^6^ cells vs 4x10^6^ cells plus irradiation (**Figure 5B**). Although the amount of *anti-*CAR CAR-T cells declined to undeletable levels in the blood of mice receiving this high cell dose at later time points, tissue infiltration remained consistent and comparable to the other groups (**Figure 5C**). Taking into account the similar kinetics of B-cell reconstitution and complete *anti-*CD19 CAR-T cell depletion, we could conclude that this condition (8x10^6^ cells, no irradiation) would be still efficient.

In summary, our findings demonstrate that administration of *anti-*CAR CAR-T cells can reverse established B-cell aplasia in a model simulating a possible therapeutic application, potentially eliminating the need for pre-conditioning before T-cell transfer.

## 4 Discussion

In this study, we investigated a cellular-based mechanism for the depletion of adoptively transferred CAR-T cells leveraging on the use of a second transgenic receptor-engineered T cell. Our concept featured a CAR targeting a short 8 amino acid peptide sequence (WSHPQFEK) called Strep-tag II, which was located in the hinge region of an *anti-*CD19 CAR. Targeting this small tag delivered a highly specific cytokine release from the *anti-*CAR CAR-T cells that ultimately translated into efficient cytotoxicity, restricted only to the *Strep*-tagged target cells. In two syngeneic mouse models of simultaneous injection of *anti-*CAR and tagged *anti-*CD19 CAR-T cells, the safeguard CAR-T cells proved functional, infiltration of secondary lymphoid organs and rapid B-cell reconstitution. More importantly, a later administration of *anti-*CAR CAR-T cells could reverse established B-cell aplasia induced by *anti-*CD19 CAR-T cells also in the absence of pre-conditioning regimes.

CAR-T cells against CD19 antigen for relapsed and refractory B-cell malignancies paved the way to cell therapy with CAR-engineered T cells. It should be considered that a significant reason for this success is due to the exclusive expression of the CD19 antigen. Being a unique B-cell lineage marker (34), side effects related to *anti-*CD19 CAR-T cells have been overall tolerable considering the clinical benefits received by patients with such advanced and aggressive tumors. Indeed, the initially life-threatening acute toxicities (CRS and CRES) (10, 11) that develop within few days after the T-cell transfer have become to a greater extent manageable (13) without any need to deplete the therapeutic living drug. In addition, new short-term manufacturing protocols for the production of CAR-T cells allowed the administration of much lower cell doses while maintaining promising clinical outcomes, presumably due to preserved T cell fitness and functionality during *ex vivo* manipulation (35, 36). As higher CAR-T cell doses usually mediate more severe side effects (37, 38), these improvements in manufacturing should further lower the risks of acute toxicities.

In contrast, on-target/off-tumor side effects due to shared tumor-associated antigens still pose a major threat, in particular for solid tumors (39, 40). Patients receiving CD19 CAR-T cells showed long-term depletion of healthy B cells and consequent hypogammaglobulinemia (5, 32). While manageable via replacement therapy, these low antibody levels could still potentially predispose CAR-treated patients to severe infections (14). Moreover, the incapacity of building *de novo* B-cell responses could limit the establishment of functional immunity against new pathogens, as observed during the SARS-CoV-2 pandemic (41). The initial expansion and persistence of CD19 CAR-T cells are crucial for achieving clinical responses (42). Still, their long-term maintenance might not be essential for sustained complete remission. Indeed, a fraction of patients with 5-year complete remission had undetectable CD19 CAR-T cells and recovered B cells already within the first 1-2 years (43). Altogether, while more studies are necessary to clarify the dynamics between CD19 CAR-T cells and endogenous B cells, the existing evidence at least raises the question of whether the maintenance of CD19 CAR-T cells is necessary after years of complete remission at the expense of a functional B-cell compartment.

Precise and complete abrogation of adoptively transferred CAR-T cells might become thus necessary to revert long-term toxicity. Among possible strategies of cell depletion in patients, the use of CAR-T cells engineered with additional suicide genes or that co-express a second targetable marker showed to be efficient but with some limitations, i.e. immunogenicity and incomplete cell eradication (21), high levels of the transgene (19), dependency on the patient’s immune system, tissue penetration and recognition of shared antigens on healthy cells (23). Similarly to others (44), we opted for a T cell-mediated recognition of tagged therapeutic CAR-T cells from a safeguard *anti-*CAR CAR-T cells, as it harbors the potential of overcoming the limitations mentioned before.

In line with existing evidence (44), we validated the feasibility of the approach *in vitro*. In addition, we carefully investigated the *in vivo* efficacy, persistence and tissue infiltration of our *anti-*CAR CAR-T cells, which would represent a unique benefit of the approach. We could show that *anti-*CAR CAR-T cells specifically recognize and eliminate *anti-*CD19 CAR-T cells in the periphery as well as in relevant secondary lymphoid organs. One of the most intriguing observation was that the transfer of *anti-*CAR CAR-T cells was efficacious also in the absence of lymphodepletion. It is believed that this step of pre-conditioning is crucial for the successful engraftment and maintenance of CAR-T cells (33), and indeed it is routinely performed in the clinic. In our data, we could confirm the beneficial effect of pre-conditioning on T-cell engraftment, as indicated by the higher frequency of circulating *anti-*CAR CAR-T cells in irradiated mice. *Anti-*CAR CAR-T cells were barely detectable (low T cell doses) or vanished (high T cell doses) in the periphery without pre-conditioning, but they reliably persisted in lymphoid organs. In line with this, we observed efficient B-cell recovery also in the absence of pre-conditioning, despite the co-existence of *anti-*CD19 CAR-T and B cells in the spleen and bone marrow of mice treated with the low cell dose. It happened also in patients receiving CD19 CAR-T cells to observe low levels of the transferred cells around the detection limit but still consistent B cell depletion (45), indicating that blood levels do not always reflect the actual persistence of CAR-T cells in disease-relevant organs. Overall, while the long-term maintenance of *anti-*CAR CAR-T cells in the absence of pre-conditioning may still be questionable, our data indicate that relatively high cell doses could still support sufficient engraftment to efficiently deplete target cells.

One limitation of the proposed approach is the slower rate of *anti*-CD19 CAR-T cell elimination compared to the use of Cetuximab. This time delay is less significant when such an approach is employed to revert long-term on-target/off-tumor toxicities that do not pose an immediate threat to the patient’s life, such as B-cell aplasia in CD19 CAR-treated patients. In these specific cases, anti-CAR CAR-T cells offer an alternative to mitigate potential safety concerns associated with Cetuximab. Indeed, as EGFR is expressed also in healthy tissues of epithelial, mesenchymal and neuronal origin, Cetuximab poses the risk of relevant toxicities e.g. skin and gastrointestinal toxicities (46). However, in situations involving life-threatening toxicities, more immediate responses are advisable. Another limitation to consider is that the persistence of *anti*-CAR CAR-T cells after antigen encounter was observed primarily at relatively high doses. Although we did not detect any signs of tonic signaling that might suggest their susceptibility to early dysfunction, further studies are needed to provide more clarity on these observations.

In summary, we demonstrated that it is feasible to generate a safeguard mechanism that exploits the specificity and sensitivity of CAR-T cells to deplete tagged therapeutic CAR-T cells. The proposed approach is generalizable to both other tags and CAR-T cells targeting different tumor-associated antigens, and might therefore be broadly applicable to enhance control over highly potent cell therapies.

## Data availability statement

The original contributions presented in the study are included in the article/**Supplementary Material**. Further inquiries can be directed to the corresponding author.

## Ethics statement

The animal study was approved by the district government of Upper Bavaria (Department 5—Environment, Health and Consumer Protection ROB- 55.2-2532.Vet_02-17-138). The study was conducted in accordance with the local legislation and institutional requirements.

## Author contributions

LW: Resources, Writing – review & editing. MS: Formal Analysis, Investigation, Methodology, Resources, Visualization, Conceptualization, Writing – original draft. SD: Resources, Writing – review & editing, Formal Analysis, Investigation, Methodology, Visualization. SF: Writing – review & editing, Resources. MT: Resources, Writing – review & editing. SR: Resources, Writing – review & editing. UJ: Resources, Writing – review & editing. ED: Conceptualization, Supervision, Visualization, Writing – original draft, Writing – review & editing. DB: Conceptualization, Funding acquisition, Supervision, Writing – review & editing.
