# Supplementary material for: A chimeric antigen receptor-based cellular safeguard mechanism for selective in vivo depletion of engineered T cells
Source: Front Immunol. 2024 Jan 11;14:1268698. doi: 10.3389/fimmu.2023.1268698 (PMC10808742; doi:10.3389/fimmu.2023.1268698)
Supplement: Supplementary file 1 [file DataSheet_1.pdf]

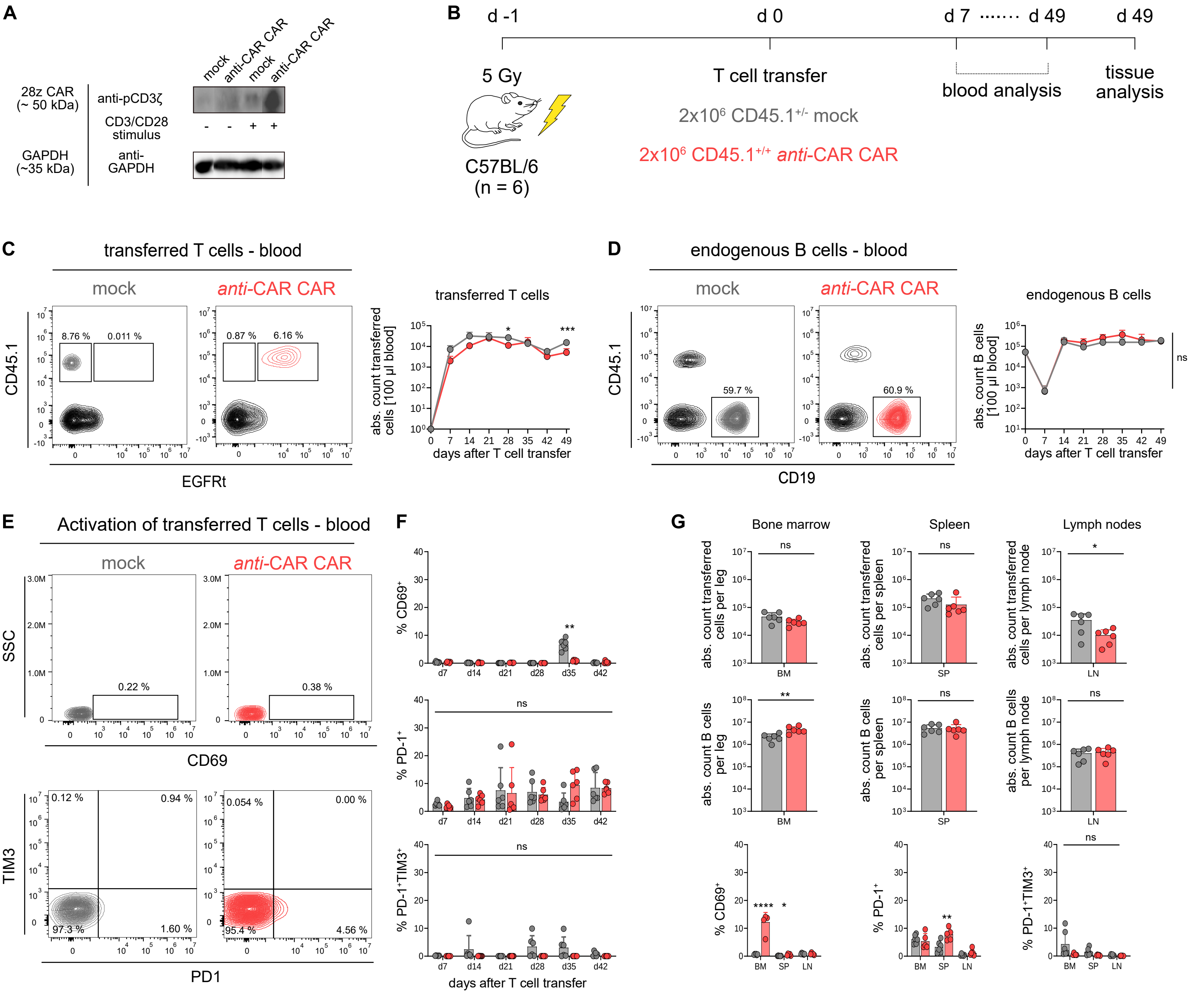

Supplementary Figure 1. Tracking of *anti*-CAR CAR-T cells *in vivo*. (A) Western blot showing pCD3ζ expression in rested or activated mock and *anti*-CAR CAR-T cells. GAPDH was used as a loading control. **(B)** Schematic of the experimental setup. **(C)** Representative flow cytometry plots (day 14 post infusion) and quantification over time of mock (CD45.1^{+}/EGFRt^{-}) and *anti*-CAR CAR-T cells (CD45.1$^{+/+}$EGFRt^{+}). **(D)** Representative flow cytometry plots and quantification of endogenous B cells over time. **(E)** Representative flow cytometry plots of mock cells and *anti*-CAR CAR-T cells expressing the activation marker CD69 and the activation/exhaustion markers PD-1 and TIM3. **(F)** Quantification of PD-1^{+}, PD1^{+}TIM3^{+} and CD69^{+} transferred T cells in blood over time. **(G)** Quantification of transferred T cells, endogenous B cells and the activation marker CD69 as well as the activation/exhaustion markers PD-1 and TIM3 in bone marrow, spleen and lymph nodes at the endpoint (day 50). Data are shown as mean + SD. In (C-G), statistical analyses were performed by two-way ANOVA with Sidak's multiple comparisons test for blood, and unpaired t-test for tissues. **p < 0.01, ***p < 0.001, *p < 0.0001.

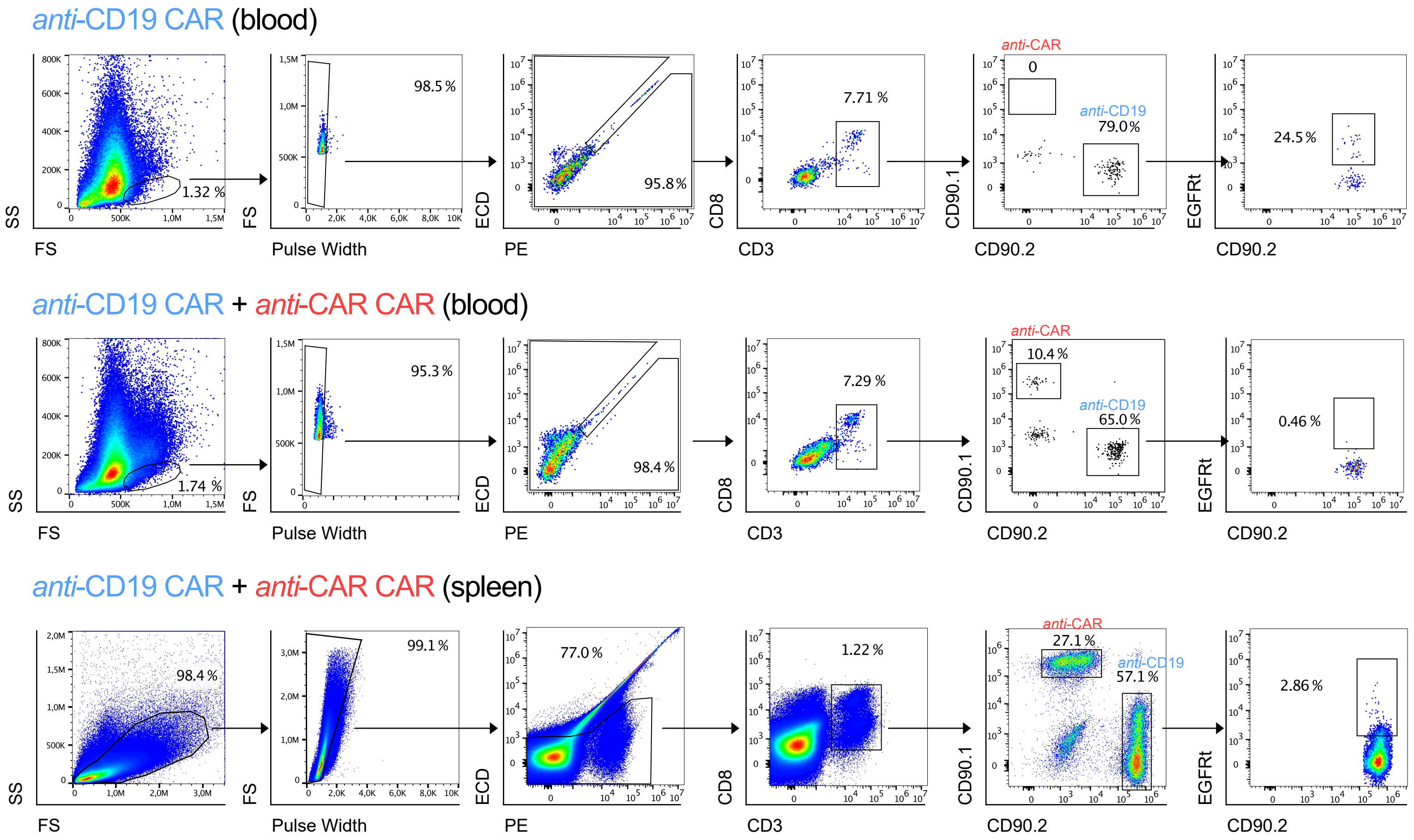

Supplementary Figure 2. Tracking of *anti*-CD19 CAR-T cells. Exemplary flow cytometry plots showing the gating strategy used to assess the persistence of *anti*-CD19 CAR-T cells (EGFRt^{+} CD90.2^{+}) according to the treatment schedule.

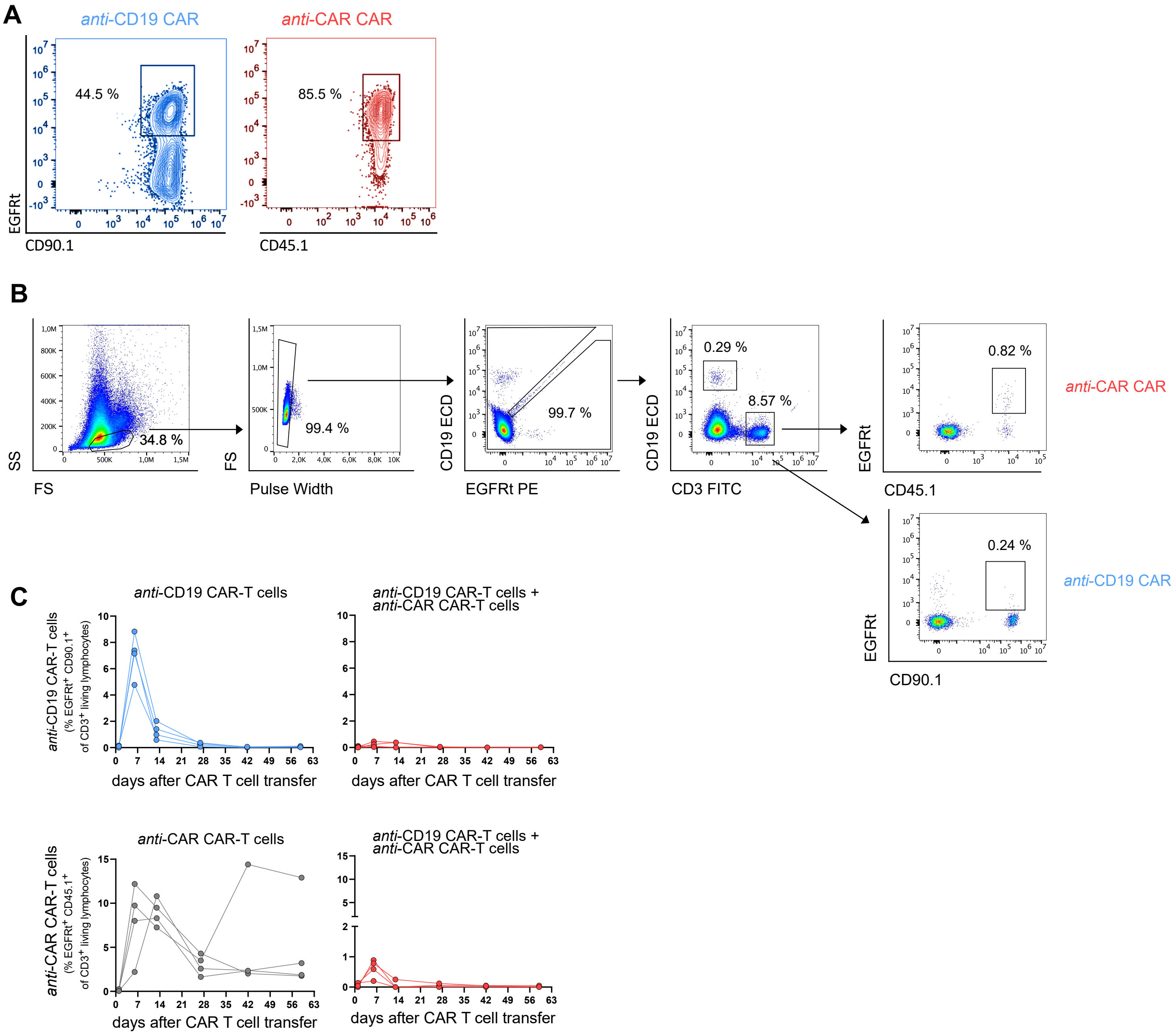

Supplementary Figure 3. Analyses of *anti*-CD19 and *anti*-CAR CAR T cells in co-transfer experiments. (A) Cell surface expression of CAR constructs after retroviral transduction. **(B)** Exemplary flow cytometry plots showing the gating strategy used to evaluate the persistence of *anti*-CD19 CAR-T cells (EGFRt$^+$ CD90.1$^+$) and *anti*-CAR CAR-T cells (EGFRt$^+$ CD45.1$^+$). **(C)** Single mice showing quantification of the frequencies of *anti*-CD19 CAR-T cells (upper) and *anti*-CAR CAR-T cells (lower) in blood over time.

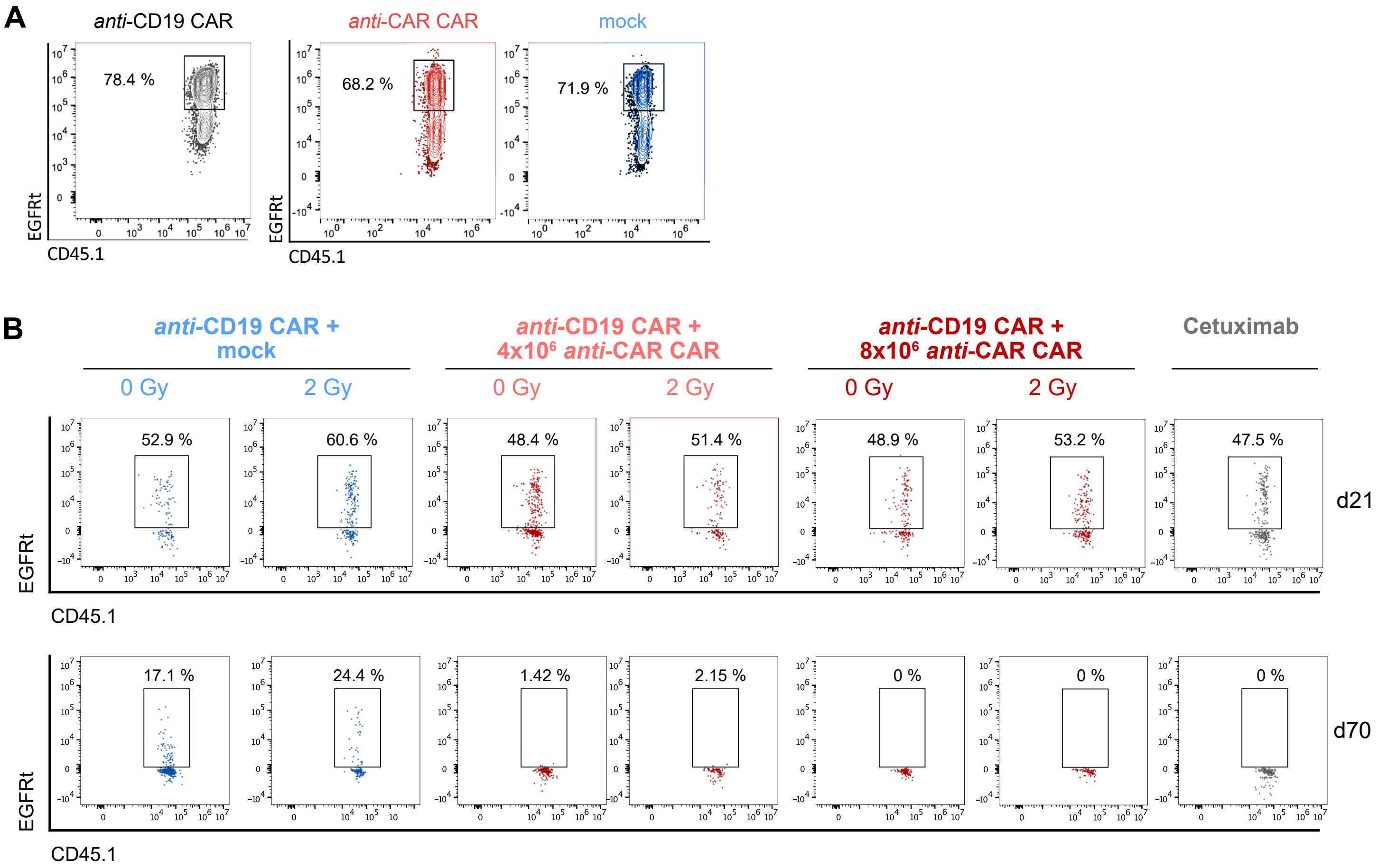

Supplementary Figure 4. Analyses of *anti*-CD19 CAR-T cells in a model of therapeutic administration of *anti*-CAR CAR-T cells. (A) Cell surface expression of CAR constructs after retroviral transduction. **(B)** Representative flow cytometry plots showing *anti*-CD19 CAR-T cells in the peripheral circulation at the indicated time points.
